# Burn Serum Increases *Staphylococcus aureus* Biofilm Formation via Oxidative Stress

**DOI:** 10.3389/fmicb.2017.01191

**Published:** 2017-06-28

**Authors:** Supeng Yin, Bei Jiang, Guangtao Huang, Yali Gong, Bo You, Zichen Yang, Yu Chen, Jing Chen, Zhiqiang Yuan, Ming Li, Fuquan Hu, Yan Zhao, Yizhi Peng

**Affiliations:** ^1^State Key Laboratory of Trauma, Burns, and Combined Injury, Institute of Burn Research, Southwest Hospital, Third Military Medical UniversityChongqing, China; ^2^Department of Microbiology, Third Military Medical UniversityChongqing, China

**Keywords:** *Staphylococcus aureus*, burn injury, serum, oxidative stress, biofilm

## Abstract

*Staphylococcus aureus* is a common pathogen isolated from burn patients that can form biofilms on burn wounds and implanted deep vein catheters, which often leads to refractory infections or even biofilm-related sepsis. As biofilm formation is usually regulated by environmental conditions, we hypothesized that serum composition may be altered after burn injury, potentially affecting the ability of infecting bacteria to form biofilms. As predicted, we observed that serum from burn-injured rats increases biofilm formation by *S. aureus* and also induces bacterial aggregation and adherence to human fibronectin and fibrinogen. Analysis of potential regulatory factors revealed that exposure to burn serum decreases expression of the quorum-sensing *agr* system and increases mRNA levels of some biofilm inducers such as *sarA* and *icaA*. In addition, we also observed that burn serum imposes oxidative stress and increases expression of key oxidoreductase genes (*sodA*, *sodM*, *katA*, and *ahpC*) in *S. aureus*. Importantly, the ability of burn serum to enhance biofilm formation and bacterial cell aggregation can be abrogated by treatment with an antioxidant. Taken together, these findings indicate that burn serum increases *S. aureus* biofilm formation via elevated oxidative stress, and may lead to novel strategies to control biofilm formation and infection in burn patients.

## Introduction

Infections are the leading cause of morbidity and mortality in burn patients (Rafla and Tredget, 2011). Damage to the skin and a compromised immune system render patients susceptible to bacterial infection. Immediately after a burn occurs, skin bacteria, respiratory and gastrointestinal flora, and environmental microorganisms may reach the wound and even enter the bloodstream, potentially leading to sepsis, multiple organ failure, and death (Church et al., 2006).

In addition to bacterial antibiotic resistance, biofilm formation is another important complicating factor in post-burn infections that often results in treatment failure and chronic infections (Kennedy et al., 2010; Metcalf and Bowler, 2013). Biofilms are organized communities of bacterial cells that are embedded in a polymeric matrix produced by the bacteria that allows them to adhere to surfaces, living as well as inanimate (Stoodley et al., 2002). The formation of biofilms is generally affected by environmental stimuli, and can serve to protect pathogens from host immune responses and antibiotics, often leading to refractory infections or even biofilm related sepsis (Costerton et al., 1999). Commonly isolated bacteria from burn patients, including *Staphylococcus aureus*, *Pseudomonas aeruginosa*, and *Acinetobacter baumannii*, often form biofilms on the ulcerated areas of burn wounds and the deep vein catheters often used in burn patients (Kennedy et al., 2010; Xiang et al., 2010).

Besides infection, complex and multi-systematic physiopathological alterations may occur simultaneously after burning. Burn serum composition reflects these changes, which include increased levels of reactive oxygen species (ROS), lipid peroxides, and some inflammatory mediators (Demling, 2005). The effects of these changes on the human body have been extensively studied (Zang et al., 2010; Corrick et al., 2015; Sun et al., 2015). Because infecting microbes often inhabit the wounds or implanted catheters of burn patients, they are also exposed to this modified environment. However, whether these changes impact bacteria is not known. We hypothesize that burn serum may affect bacterial survival and pathogenesis-related functions such as biofilm formation.

*Staphylococcus aureus* is one of the most common pathogens isolated from burn patients (Yali et al., 2014). The high prevalence of multiple drug-resistant *S. aureus* at burn centers, and its tendency to form biofilms, presents a difficult challenge for clinicians. Biofilm formation of *S. aureus* is often regulated by some stress conditions, such as temperature, sodium chloride, glucose, and oxidative stress (Rode et al., 2007). These stimuli further affect the complicated pathways involved in biofilm formation including both *ica*-dependent and *ica*-independent mechanisms. Among these pathways, the *ica* operon and some global regulators such as *agr* system and *sarA* are widely studied (Laverty et al., 2013).

In this study, we used *S. aureus* as a model organism and focused on its ability to form biofilms in the presence of burn serum. We observed that exposure of *S. aureus* to serum from thermally injured rats increases staphylococcal biofilm formation both *in vitro* and *in vivo*. Burn serum also enhances bacterial adherence to human fibronectin and fibrinogen, thereby promoting aggregation of *S. aureus* cells. Exposure of *S. aureus* to burn serum also markedly increases the transcription of some oxidoreductase genes. Treatment with antioxidant abrogates the ability of burn serum to increase *S. aureus* biofilm formation and cell aggregation. Together, these observations suggest that enhanced biofilm formation by *S. aureus* in burn serum is due to elevated oxidative stress. To our knowledge, this is the first report on the relationship between burn serum and *S. aureus* biofilm formation. These findings may provide a foundation for novel strategies to control biofilms and infections in burn patients.

## Materials and Methods

### Bacterial Strains and Culture Conditions

*Staphylococcus aureus* strains Newman, ATCC25923, N315, and two clinical isolates SAO1 and SAO2 (obtained from the Institute of Burn Research, Southwest Hospital, Chongqing, China) were used in the present study. Unless otherwise stated, all strains were grown in tryptic soy broth (TSB) at 37°C with shaking at 200 rpm. Assays for biofilm formation were conducted in TSB supplemented with 0.5% glucose.

### Burn Procedure and Burn Serum Isolation

Full-thickness cutaneous burns covering 40% total body surface area (TBSA) in rats were generated as previously described (Zang et al., 2010). Briefly, male SD rats (250–300 g) were anesthetized with amobarbital sodium and shaved before injury. Then they were placed in a device that left approximately 40% of their body surface area exposed. Rats were given full thickness scald burns by immersion in 100°C water for 12 s. Sham-burned rats were shaved and placed in water at room temperature for 12 s. All rats were warmed and supplemented intraperitoneally with lactated Ringer’s solution (4 ml/kg per percent burn), according to the Parkland burn formula. Twenty four hours post-burn, rats were euthanized to obtain serum samples, which were then used immediately for biofilm formation assays or chemical analysis.

### Biofilm Formation in 96-Well Microtiter Plates

Microtiter plate assays were performed as described earlier with modifications (Kulkarni et al., 2012). Overnight *S. aureus* cultures were diluted 1:100 in medium containing various concentrations (0–50% vol/vol) of burn or sham serum in a 96-well plate and incubated for 24 h on a rocker at 37°C. When required, 10 mM L-ascorbic acid (AA) was added to the cultures. After incubation for 24 h, the plate was rinsed gently with deionized water three times to remove planktonic bacteria. The remaining biomass was fixed by baking at 60°C for 1 h, followed by staining with 0.3% crystal violet for 15 min, and then rinsed with running tap water to remove unbound stain. Finally, the plate was dried and the dye bound to the adherent biomass was extracted in 100 μl 70% ethanol–10% methanol mixture. The optical density of the extract was determined at 590 nm (OD_590_).

### Confocal Laser Scanning Microscopy (CLSM)

An overnight culture of *S. aureus* strain ATCC25923 was diluted 1:100 in TSB medium with 50% burn or sham serum in 15 mm glass-bottom cell culture dishes (polystyrene) and cultured for 24 h on a rocker at 37°C. The dishes were then rinsed gently with deionized water three times and fixed with 4% formaldehyde. A 2-mL aliquot of the red fluorescent nucleic acid stain SYTO 61 (Invitrogen), diluted 1:1000 in PBS, was added to the dishes, followed by incubation in the dark at room temperature (Hammond et al., 2010). After 30 min, the dishes were drained, rinsed with deionized water, and 2 ml of a solution of 50 μg/ml FITC-labelled concanavalin A type IV (Sigma–Aldrich) was added in order to stain extracellular polysaccharide green (Aslam and Darouiche, 2011). After 5 min of incubation in the dark at 37°C, the dishes were rinsed with deionized water and air-dried. CLSM images were acquired using a laser scanning confocal microscope (LSM780, Carl Zeiss) equipped with a Plan-Apochromat 63×/1.40 Oil M27 objective lens. The excitation wavelength was 561 nm and emission was 640 nm for SYTO61, while for FITC-ConA they were 488 and 537 nm, respectively. Images were analyzed and processed using the ZEN image analysis package (Carl Zeiss). Three independent experiments were performed. All images were acquired from at least three distinct regions of the cell culture dishes and representative ones were selected.

### Quantitative Real-Time PCR

*Staphylococcus aureus* Newman and ATCC25923 were challenged with 50% burn or sham serum and grown to late exponential phase. Total RNA was isolated using TriPure RNA isolation reagent (Roche) and reverse transcribed to cDNA using a first-strand cDNA synthesis kit (Thermo Fisher Scientific). qRT-PCR was then performed in a 7500 real-time PCR system (Applied Biosystems) using SYBR green real-time PCR master mix (TOYOBO). 16S rRNA was used as an internal control. Genes and their corresponding qRT-PCR primers are listed in Supplementary Table S1. Fold changes in gene transcript levels were quantified using the comparative threshold cycle (ΔΔC_T_) method. Results are expressed as relative fold changes in transcript levels in bacteria cultured in burn serum, compared to the values observed for bacteria cultured in sham serum, with the latter normalized to a value of 1.

### Biochemical Analyses of Burn and Sham Serum

The biochemical alterations in burn serum were examined immediately after collection. Levels of malondialdehyde (MDA), glutathione peroxidase (GPX3), and superoxide dismutase (SOD3) were measured using commercial assay kits (Cloud-Clone Corp.) in accordance with the manufacturer’s recommendations. Aliquots of serum samples were submitted to the clinical laboratory at Southwest Hospital to measure concentrations of glucose, sodium ions, and chloride ions.

### Aggregation Assay

Aggregation assays were conducted as described previously (McCourt et al., 2014). Briefly, *S. aureus* strains exposed to 50% burn serum, sham serum, or TSB medium were cultured overnight with and without the treatment of AA. A few microliters was withdrawn from each culture, stained with crystal violet, and observed by light microscopy. In parallel, one milliliter of medium was removed from the top of each culture, the OD_600_ was measured and recorded as OD_600_-1. The remaining culture was vortexed to separate aggregated cells, and the OD_600_ was measured again (OD_600_-2). The percentage of aggregation was calculated as: 100 × [(OD_600_–2–OD_600_–1)/OD_600_–2].

### Bacterial Adherence to Human Fibronectin and Fibrinogen

Human fibronectin and fibrinogen (Sigma) were diluted in 100 μl 0.05 M Na_2_CO_3_–NaHCO_3_ coating buffer and added to 96-well plates overnight at 4°C to allow the proteins to adhere to the plastic substrate (Ythier et al., 2012; McCourt et al., 2014). After washing, wells were blocked with 1% bovine serum albumin (BSA). Bacteria cultured overnight in medium containing 50% burn serum, 50% sham serum, or TSB alone, and with or without 10 mM AA, were harvested by centrifugation. Bacteria were resuspended in PBS and the concentration was adjusted to an OD_600_ of 1.0. 100 μl of the resuspended cells were added to each well and incubated for 2 h at 37°C. The wells were then washed with PBS, and adherent bacteria were fixed at 60°C and stained with crystal violet as described above.

### Bacterial Colonization on Central Venous Catheters

Male SD rats (250–300 g) were subjected to 40% TBSA burn or sham-burn injury as described above. Central venous catheters were then implanted as described by Ebert et al. (2011) with modifications. The necks of rats suffering burn or sham injury were shaved and the skin was sterilized with an iodophor disinfectant. A 1 cm incision was made on the right side of the neck to expose the right jugular, which was then fixed by a 5–0 silk suture. The catheter was inserted into the jugular vein by making a micro-incision and advanced caudally about 2 cm to the vena cava. Correct positioning was verified by blood withdrawal, and then heparin-locking solution was injected. Finally, the catheter was held in place and the skin was closed by 3–0 silk suture. Twenty-four hours later, all the rats were inoculated with 10^6^ CFU of *S. aureus* ATCC25923 via the tail vein. Half of the burn and sham injured rats were selected randomly to receive AA (100 mg/day) intraperitoneally for 7 days (*n* = 12 rats per group). Control rats received the same volume of saline. Blood was harvested through the tail vein at 3, 5, and 7 days post-infection, and bacterial load was evaluated. At 7 days post-infection, the rats were sacrificed. The catheters were removed and rinsed three times with deionized water to wash out planktonic bacteria. Each catheter was placed in 1 ml of PBS and subjected to ultrasonic agitation (45 kHz, 100% Power) for 5 min to detach sessile bacteria from the biofilms formed on the catheters. The fluid was serially diluted and viable bacteria were counted using the drop plate method.

### Ethics Statement

The animal experiments in this study were performed in accordance with the International Guiding Principles for Biomedical Research involving Animals-1985 and approved by the Laboratory Animal Welfare and Ethics Committee of Southwest Hospital, Third Military Medical University.

### Statistical Analysis

Data were analyzed by one-way analysis of variance (ANOVA) or Student’s unpaired *t*-test as appropriate, using GraphPad Prism analysis package. A *P*-value of less than 0.05 was considered statistically significant.

## Results

### Burn Serum Challenge Increases Biofilm Formation by *S. aureus*

**FIGURE 1:**
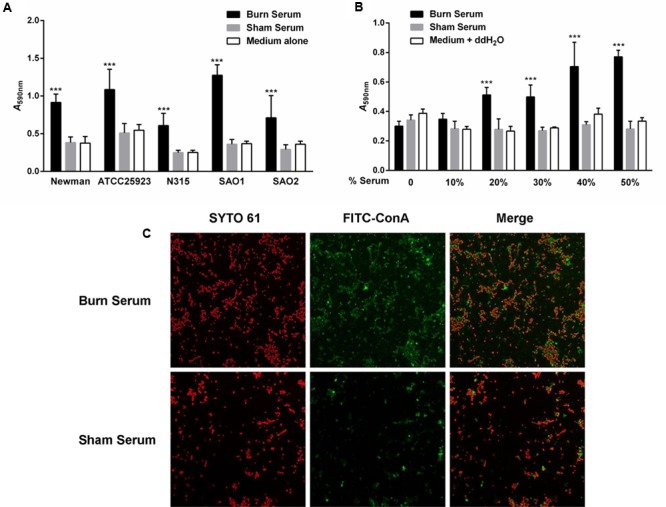
Burn serum exposure increases biofilm formation of *Staphylococcus aureus*. **(A)** The indicated strains of *S. aureus* were exposed to 50% burn serum, 50% sham serum or TSB medium alone in a 96-well plate for 24 h. **(B)** *S. aureus* Newman was exposed to various concentrations of burn serum, sham serum or ddH_2_O for 24 h. For both **(A,B)**, biofilm biomass was stained with crystal violet and absorbance of the extracted dye was measured at 590 nm. Values are shown as means ± standard deviations and represent three independent experiments. ^∗∗∗^*P* < 0.001 indicates significant differences between burn serum-challenged samples and sham serum-treated counterparts or control. **(C)** CLSM images (×630) of biofilms formed by *S. aureus* ATCC25923 in medium supplemented with 50% burn or sham serum. Biofilms were stained with a red fluorescent nucleic acid stain (SYTO 61) to visualize bacteria, and a green fluorescent biofilm matrix stain (FITC-ConA) to visualize extracellular polysaccharide.

To determine the effect of burn serum on biofilm formation, multiple strains of *S. aureus* were exposed to burn or sham serum collected from SD rats, and biofilm formation was quantified using a microtiter plate assay. Burn serum significantly increased biofilm formation in *S. aureus* strains Newman, ATCC25923, N315 and two clinical isolated strains SAO1 and SAO2 (**Figure 1A**). Increasing concentrations (0 to 50%) of burn serum resulted in a dose-dependent increase in biofilm formation in Newman (**Figure 1B**).

To confirm these results, we used CLSM to observe biofilm formation of *S. aureus* ATCC25923 cultured in burn and sham serum. Bacterial cells were then stained red with SYTO 61 and extracellular polysaccharide was stained green with FITC-labeled concanavalin A. Exposure to burn serum results in a denser and more compact florescent biomass (**Figure 1C**). This confirms that burn serum enhances the accumulation of both bacterial cells and extracellular polysaccharide, consistent with the results from the microtiter plate assay.

### Burn Serum Challenge Alters the Transcription of Genes Involved in Biofilm Formation

Multiple molecules and regulatory factors have been implicated in staphylococcal biofilm structuring and dispersal. To identify the pathways that are activated in *S. aureus* upon exposure to burn serum, transcript levels for 14 representative genes from several pathways were measured. Because the expression of some biofilm-associated genes showed very low levels in the growth of early and mid-exponential phases, we finally tested the mRNA levels in late exponential phase.

**FIGURE 2:**
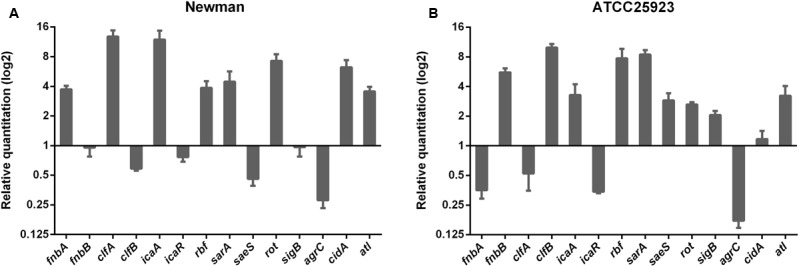
Burn serum challenge alters the transcription of genes involved in biofilm formation. mRNA levels of biofilm-associated genes in *S. aureus* Newman **(A)** and ATCC25923 **(B)** were quantified by qRT-PCR after strains were challenged with 50% burn serum or sham serum. Fold changes were calculated using the threshold cycle (ΔΔC_T_) method. The bars represent relative fold changes for genes in burn serum-treated strains compared to their sham serum-treated counterparts (normalized as 1). The data are from triplicate readings in one representative experiment of three independent trials.

As shown in **Figure 2**, mRNA levels for the surface adhesin genes, *fnb* (fibronectin binding protein), and *clf* (clumping factor), exhibit strain-specific responses. In Newman, levels of *fnbA* and *clfA* mRNA increased, while in ATCC25923, levels of *fnbB* and *clfB* increased. Transcript levels for most of the positive regulators that are involved in the *ica*-dependent pathway, such as *icaA* (intercellular adhesin A), *rbf* (required for biofilm formation), and *sarA* (staphylococcal accessory regulator A) increased in both Newman and ATCC25923. This was also the case for independent regulators such as *atl* (autolysin-encoding gene) and *rot* (repressor of toxins). In contrast, levels of *saeS* (in the *sae* operon) and *sigB* (sigma B) mRNA increased only in ATCC25923, while levels of *cidA* (in the *cid* operon) increased only in Newman. Transcripts for the negative regulators *agrC* (accessory gene regulator C) and *icaR* decreased in both strains, although *icaR* showed only a modest decrease (1.3-fold) in Newman. In addition, the transcription of alphatoxin (*hla*) also decreased when challenged with burn serum (data not shown), which confirmed the down-regulation of *agr* system. Overall, the majority of genes associated with biofilm formation have similar expression patterns in Newman and ATCC25923.

### Burn Serum Induced Biofilm Formation is a Response to Increased Oxidative Stress

Environmental factors, including levels of glucose, sodium chloride, and some stress conditions such as oxidative stress, play important roles in the regulation of staphylococcal biofilm formation (Rode et al., 2007; Kulkarni et al., 2012). To identify whether burn serum might impose one or more of these stress factors, we compared levels of glucose, NaCl, and oxidative stress factors in burn and sham sera. Burn serum showed only a slightly decrease (with no significant differences) in levels of glucose, sodium ions, and chloride ions (**Supplementary Figure S1**). However, glucose and NaCl levels are positively correlated with staphylococcal biofilm formation in these concentration ranges (Lim et al., 2004; You et al., 2014). Thus, changes in glucose and NaCl do not explain the ability of burn serum to promote biofilm formation.

**FIGURE 3:**
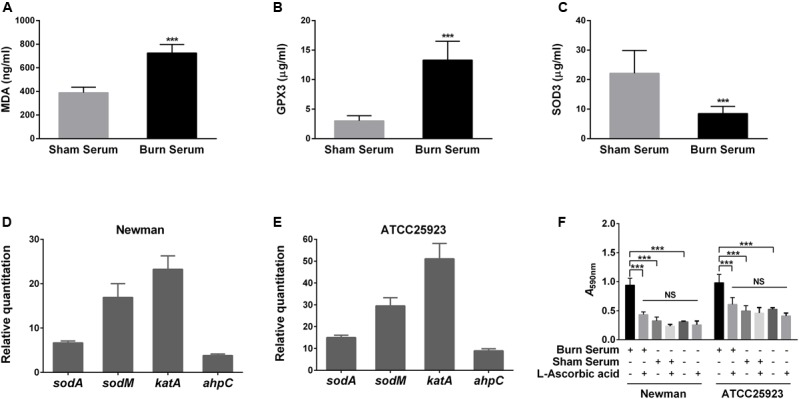
Burn serum induces biofilm formation via oxidative stress. Levels of MDA **(A)**, GPX3 **(B)**, and SOD3 **(C)** were measured in sera obtained from SD rats 24 h after suffering a 40% TBSA burn or sham-burn injury. ^∗∗∗^*P* < 0.001 indicates differences are significant relative to levels in sham serum. **(D,E)** mRNA levels for genes responding to oxidative stress in *S. aureus* after challenge with 50% burn serum or sham serum were determined by qRT-PCR. Transcript levels for genes in the sham serum-treated strains were normalized to 1. **(F)** Treatment with antioxidant L-ascorbic acid abrogated the ability of burn serum to enhance biofilm formation. ^∗∗∗^*P* < 0.001 indicates differences are significant between burn serum-treated strains and those treated otherwise. Data are presented as mean ± standard deviations from three independent trials.

Following a severe burn injury, numerous free radicals and ROS are produced as the result of increased xanthine oxidase and neutrophil activation (Horton, 2003; Parihar et al., 2008). The elevated oxidative stress causes lipid peroxidation and induces antioxidant defenses in the host. We therefore evaluated the MDA content, which is a marker of lipid peroxidation, and the level of antioxidant enzymes, such as glutathione peroxidase (GPX3) and superoxide dismutase (SOD3), in burn and sham sera. As expected, MDA and GPX3 levels were significantly increased in burn serum (**Figures 3A,B**), while SOD3 levels were remarkably reduced (**Figure 3C**), consistent with previous studies (Kumar et al., 1995; Cetinkale et al., 1997). These data demonstrate that factors contributing to oxidative stress are elevated in burn serum.

Next, to examine whether the elevated oxidative stress imposed by burn serum is responsible for biofilm formation by *S. aureus*, we analyzed the expression of antioxidant enzymes in the Newman and ATCC25923 strains. qRT-PCR analysis shows that mRNA levels for the oxidoreductase genes *sodA*, *sodM*, *katA* (catalase), and *ahpC* (alkyl hydroxy peroxidase) increased significantly in both strains (**Figures 3D,E**), indicating a strong response to oxidative stress. For further confirmation, we pretreated burn serum with the antioxidant L-ascorbic acid (AA) to eliminate excessive ROS. Treatment with AA abrogated the ability of burn serum to increase biofilm formation, while AA itself does not affect biofilm formation significantly in the absence of burn serum (**Figure 3F**). Taken together, these data suggest that oxidative stress imposed by burn serum promotes staphylococcal biofilm formation.

### Burn Serum Induces Bacterial Aggregation and Adherence to Human Fibronectin and Fibrinogen

**FIGURE 4:**
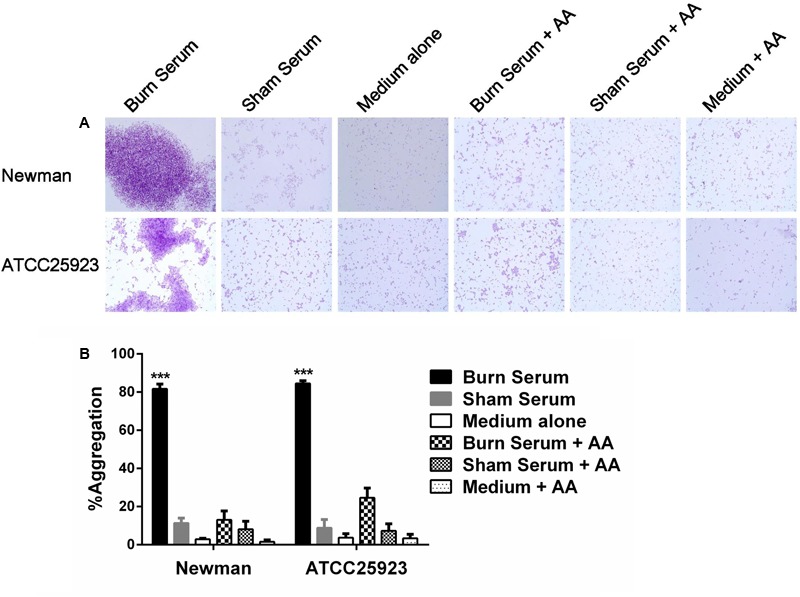
Burn serum promotes bacterial aggregation. *S. aureus* strains were cultured in broth containing 50% burn serum, 50% sham serum, or TSB medium alone, with or without overnight treatment with antioxidant. **(A)** Bacteria were stained with crystal violet and observed by light microscopy (×1000). Images shown are representative of at least three distinct regions. **(B)** The percentage aggregation values were calculated. ^∗∗∗^*P* < 0.001, indicates differences are significant between burn serum-treated strains and those treated otherwise. The data were from three independent experiments.

Since surface adhesin gene transcripts are more abundant in *S. aureus* after exposure to burn serum, we hypothesized that aggregation by *S. aureus* may also increase. As predicted, microscopy revealed that the bacterial cells tend to aggregate in the presence of burn serum during growth (**Figure 4A**). Aggregation of *S. aureus* exposed to burn serum in stationary phase increased substantially in comparison with controls (**Figure 4B**). Consistent with the results described above, treatment with AA blocks this response (**Figure 4**).

**FIGURE 5:**
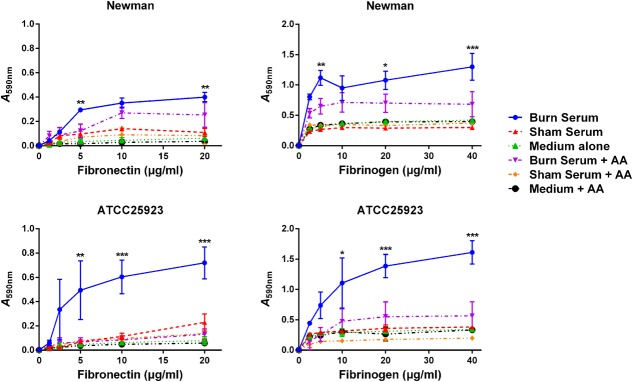
Burn serum enhances bacterial adherence to human fibronectin and fibrinogen. *S. aureus* Newman and ATCC25923 overnight cultures were subjected to the same growth conditions as described for **Figure 4**, and were centrifuged and resuspended in PBS. Bacterial suspensions were then added to wells coated with fibronectin and fibrinogen. Adherent cells were stained with crystal violet and the absorbance of the extracted dye was determined at 590 nm. Values represent results from three independent experiments. ^∗^*P* < 0.05, ^∗∗^*P* < 0.01, and ^∗∗∗^*P* < 0.001 indicate differences are significant between burn serum-treated strains and those treated otherwise.

To extend this result, *S. aureus* in stationary phase was exposed overnight to burn or sham serum, and then analyzed in solid phase assays to measure adherence to immobilized human fibronectin and fibrinogen. Adherence of Newman and ATCC25923 was significantly enhanced by growth in burn serum. The effect was eliminated by AA (**Figure 5**). These data indicate that increased levels of surface adhesins increase bacterial adherence to human fibronectin and fibrinogen, thereby promoting aggregation of *S. aureus* cells.

### Burn Injury Increases Colonization of *S. aureus* on Implanted Central Venous Catheters *In Vivo*

**FIGURE 6:**
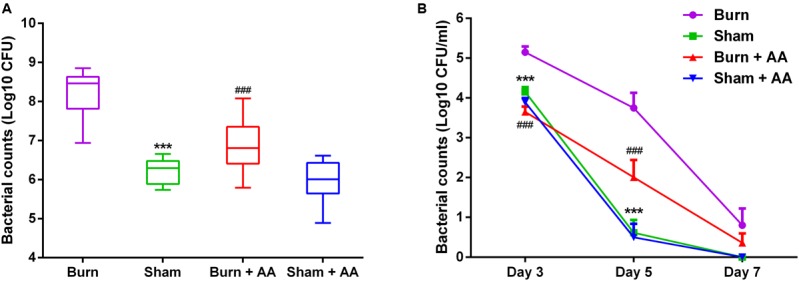
Burn injury increases bacterial colonization on implanted central venous catheters. A total of 48 male SD rats implanted with central venous catheters were inoculated with 10^6^ CFU of *S. aureus* ATCC25923 24 h after burn or sham injury. Half of the rats in each group were randomly selected to receive either AA (100 mg/day) or saline intraperitoneally for 7 days (*n* = 12 rats per group). One rat in the burn group died 1 day after inoculation. Blood was harvested at 3, 5, and 7 days post-infection and bacterial loads were evaluated **(B)**. All the rats were sacrificed at 7 days post-infection and the number of bacteria colonized on the catheters was assessed **(A)**. ^∗∗∗^*P* < 0.001, ^###^*P* < 0.001 compared with burn rats. These data are from one representative experiment of three independent replicates.

To investigate the effect of burn serum on biofilm formation *in vivo*, we implanted central venous catheters in adult rats and challenged them with *S. aureus* ATCC25923 24 hours after burn or sham injury. Half of the burn and sham injured rats (*n* = 12 in each group) were treated with a high dose of AA (100 mg/day). One rat in the burn group died 1 day after inoculation. Seven days after infection, counts of viable bacteria attached to implanted catheters were significantly higher in burn rats than sham rats, or in burn rats treated with AA (**Figure 6A**). Interestingly, the bacterial loads in blood from burn rats on days 3 and day 5 after infection were also much higher than the loads in sham rats or AA-treated burn rats (**Figure 6B**). AA did not significantly affect bacterial loads or colonization in sham rats. These results suggest that burn injury increases *S. aureus* colonization on implanted central venous catheters *in vivo* and compromises bacterial clearance in rats. Moreover, administration of the antioxidant AA can alleviate these effects.

## Discussion

Biofilm formation constitutes a major threat to patients with indwelling devices or chronic infections (Donlan and Costerton, 2002; Metcalf and Bowler, 2013; Romling et al., 2014). It is generally known that burn patients are particularly susceptible to infections. Infecting bacteria can also form biofilms in burn wounds and implanted deep vein catheters (Kennedy et al., 2010; Xiang et al., 2010). Because biofilm formation is often regulated by environmental stimuli, we hypothesized that burn injuries may alter serum chemistry in a way that affects the ability of infecting bacteria to form biofilms. In this study, we demonstrated that serum from burn-injured rats enhances biofilm formation by *S. aureus*, one of the most common pathogens that infects burn patients.

Because serum and serum components can inhibit biofilm formation in some bacteria (Ardehali et al., 2003; Hammond et al., 2010; Ding et al., 2014; Wuren et al., 2014), the potentiation of biofilm formation by burn serum must be due to alterations in the serum after burn injury. After excluding several factors that can affect biofilm formation, we focused on the elevation of factors related to oxidative stress in burn serum. Two key observations are consistent with oxidative stress as a triggering mechanism: burn serum stimulates the expression of oxidoreductase genes in *S. aureus*, and the ability of burn serum to enhance biofilm formation is abrogated by antioxidants. Numerous ROS are produced after burn injury as the result of increased xanthine oxidase activity and neutrophil activation. Elevated levels of free radicals and ROS can cause an inflammation response in the host, leading to tissue damage and multiple organ failure (Horton, 2003; Parihar et al., 2008). Bacteria that infect the burned host are also exposed to the potentially damaging effects of ROS. Sublethal levels of hydrogen peroxide or ROS can enhance biofilm formation. Antunes et al. (2012) and Kulkarni et al. (2012) reported elevated oxidative stress in smoke induced biofilm growth in *S. aureus* and *P. aeruginosa*. Treatment of an alkyl hydroperoxide reductase (*ahpC*) mutant with antioxidants reduces biofilm formation by *Campylobacter jejuni*, indicating that accumulated ROS induces biofilm formation (Oh and Jeon, 2014). Moreover, ROS, RNI (reactive nitrogen intermediates) and their downstream derivatives play an important role in staphylococcal biofilm development (Arce Miranda et al., 2011). These reports, in combination with our data, support the conclusion that the increase in ROS in burn serum is the major factor that promotes biofilm formation by *S. aureus*.

The molecular mechanisms of staphylococcal biofilm formation are highly complex. The regulatory pathways constitute an intricate network of overlapping circuits (Archer et al., 2011; Laverty et al., 2013). In this study, we found that many pathways and regulators are activated when *S. aureus* is exposed to the elevated ROS in burn serum, including the *ica*-dependent pathway, factors controlling eDNA release (*cidA* and *atl*), regulators affecting extracellular protease or nuclease activity (*sarA, saeS, rot, sigB*, and *agrC*), and the oxidoreductase genes responding to oxidative stress (*sodA, sodM, katA*, and *ahpC*). Among these, the quorum-sensing *agr* system is coupled in some way with oxidation sensing. The transcription of the *agr* system decreases in *S. aureus* that has been challenged with cigarette smoke (Kulkarni et al., 2012). Oxidative stress can induce disulfide bond formation in AgrA and, in turn, reduce *agrC* transcription (Sun et al., 2012). These results are consistent with our observation that mRNA levels of *agrC* and its downstream gene *hla* decrease markedly in *S. aureus* under the oxidative stress imposed by burn serum. Importantly, the *agr* system is a global regulator in *S. aureus*, and many other regulators such as *sarA*, *sae*, *rot*, and *sigB* have interconnections with the *agr* system (Lauderdale et al., 2009; Archer et al., 2011; Johnson et al., 2011; Mootz et al., 2015). Therefore, we speculate that the oxidative stress that occurs upon exposure to burn serum affects the bacterial cell mainly through negative regulation of the *agr* system, subsequent activation of other global regulators and pathways, and ultimately the overexpression of surface adhesins that enhance cell aggregation and biofilm formation. However, the mechanisms by which the *agr* system is affected and then regulates other pathways need to be further elucidated.

The enhanced bacterial aggregation and adherence to fibronectin and fibrinogen are primarily due to the overexpression of surface adhesins, which are under the control of global regulators such as *agr* and *sarA* (Archer et al., 2011). The alterations we detected in *agrC* and *sarA* transcription are consistent with observations that the *agr* locus is down-regulated when *S. aureus* adheres to fibrinogen, while the *sar* locus is up-regulated to enable adherence to fibronectin (Shenkman et al., 2001). Enhanced cell aggregation and binding ability are adaptive survival strategies for bacteria responding to environmental stress, although these changes may lead to invasive and refractory infections in the host.

Finally, in the experimental animal infection model, we observed that burn injury increases *S. aureus* colonization on implanted central venous catheters, and this effect can be alleviated by treatment with high doses of AA, a conventional anti-oxidant therapy in burn injury. This result is consistent with our *in vitro* study. Burn injury also compromises bacterial clearance in rats in the early stage of infection, and AA treatment accelerates bacterial clearance in burn rats as well. These observations are in accordance with a recent study showing that AA supplementation attenuates hyperoxia-compromised host defenses against pulmonary bacterial infection by scavenging excessive ROS (Patel et al., 2016). Taken together, these data suggest that the administration of AA can help the host control bacterial infection and biofilm formation in several ways, although it is not yet possible to determine which mechanism is more important *in vivo*. Therefore, in addition to the demonstrated effects of antioxidant therapy in preventing tissue or cell damage caused by ROS (Matsuda et al., 1993; Jewo et al., 2012; Oudemans-van Straaten et al., 2014), antioxidant therapy may also be a strategy for helping antibiotics control bacterial infection and biofilm formation in burn injury.

## Author Contributions

The author(s) have made the following declarations about their contributions: YP, YZ, and SY conceived and designed this study; SY, BJ, GH, BY, ZY, YG, and YC performed the experiments; SY, JC, ZY, and FH analyzed the data; ML, YZ, YP, and SY drafted the manuscript.

## Conflict of Interest Statement

The authors declare that the research was conducted in the absence of any commercial or financial relationships that could be construed as a potential conflict of interest.

## Supplementary Material

Figure S1 Concentration of glucose and sodium chloride in burn serum and sham serum. The concentration of glucose, sodium ions, chloride ions in the serum obtained from the SD rats 24 h after suffering a 40% TBSA burn or sham-burn injury was immediately measured by biochemical examination. Data are from two separated experiments.
