# Supplementary material for: Burn Serum Increases Staphylococcus aureus Biofilm Formation via Oxidative Stress
Source: Front Microbiol. 2017 Jun 28;8:1191. doi: 10.3389/fmicb.2017.01191 (PMC5487419; doi:10.3389/fmicb.2017.01191)
Supplement: Supplementary file 2 [file Data_Sheet_1.docx]

**Appendix A: Buddhist Identification Questionnaire**

Instead of subjects’ self-reports of being a Buddhist, we designed the following questionnaire to verify our Buddhist group:

1. When did you start to accept a Buddhist worldview?

2. Do you believe in reincarnation?

3. Do you agree with the following statement: “All sufferings in life are due to one’s ignorance of the real causal mechanism regarding how things work; thus, erroneous opinions, words, and behaviors are produced”?

4. Do you believe that one can understand the nature (ontology) of the universe?

5. Do you agree with the following statement: “External things do not exist. They are but the work of our mind, like a painting to the painter”?

6. Which school/sect of Buddhist worldview do you subscribe to? (Please list according to the order of “I agree with most” to “I agree with least.”)

7. Which literature do you think most effectively depicts the truth according to Buddhism? (Please list according to the order of “I agree with most” to “I agree with least.”)
